# Sequenzvarianten unklarer Signifikanz bei Small-Fiber-Neuropathie: Charakterisierung einer heterogenen PatientInnenpopulation

**DOI:** 10.1007/s00482-024-00811-3

**Published:** 2024-05-07

**Authors:** Caren Meyer zu Altenschildesche, Nadine Egenolf, Annette Lischka, Nurcan Üçeyler

**Affiliations:** 1 Neurologische Klinik des Universitätsklinikums Würzburg, Josef-Schneider-Str. 11, 97080 Würzburg, Deutschland; 2 Institut für Humangenetik und Genommedizin, Universitätsklinikum RWTH Aachen, Aachen, Deutschland

**Keywords:** Schmerz, Genetik, Schmerzgene, Schmerzsyndrom, Kleinfaserneuropathie, Pain, Genetics, Pain genes, Pain syndrome, Small fiber neuropathy

## Abstract

**Hintergrund:**

Bei etwa der Hälfte der PatientInnen mit Small-Fiber-Neuropathie (SFN) findet sich für die Schmerzsymptomatik keine erklärende und behandelbare Ätiologie. Es häufen sich Berichte zu genetisch-neuropathischen Schmerzsyndromen. Auch einige SFN-PatientInnen weisen Variationen in schmerzassoziierten Genen auf. Teils sind diese bereits als „pathogen“ bestätigt, andere haben eine „unklare pathogenetische Relevanz“. Trotz des hohen Anteils betroffener PatientInnen ist über die genetische SFN bislang wenig bekannt.

**Fragestellung:**

In unserer Arbeit fokussierten wir uns auf diese Kohorte: Durch Sammlung klinischer Daten sollten Charakteristika der PatientInnen mit seltenen Varianten unklarer Signifikanz in schmerzassoziierten Genen gesammelt werden.

**Material und Methoden:**

Von 2015 bis 2020 untersuchten wir 66 PatientInnen mit initial „idiopathischer“ SFN. Bei 13/66 (20 %) wurden Varianten unklarer pathogenetischer Relevanz in schmerzassoziierten Genen detektiert. Alle rekrutierten PatientInnen unterzogen sich einer detaillierten Anamneseerhebung mit Fokus auf Schmerz und beantworteten Fragebögen zu Beschwerden und Belastung.

**Ergebnisse:**

Die Kohorte mit seltener Variante in schmerzassoziierten Genen zeigte gegenüber den anderen PatientInnen subtile klinische Unterschiede: Neben einer höheren physischen und psychischen Belastung konnten eine von außen beeinflussbare Symptomatik und eine herausfordernde Therapie nachgewiesen werden.

**Diskussion:**

Wir sehen eine frühzeitige genetische Diagnostik bei SFN als essenziell: Durch weitere supportive Maßnahmen wie Vermeidung von Einflussfaktoren, Stärkung der Resilienz und eventuell künftig verfügbare zielgerichtete Therapeutika kann die Versorgung der PatientInnen mit seltener Variante in schmerzassoziierten Genen optimiert werden.

## Abstract (en)

**Background:**

In almost half of patients suffering from small fiber neuropathies (SFN), the etiology remains elusive. For these patients with “idiopathic SFN”, symptomatic analgesic therapy is the only option. Reports on a potential genetic background of neuropathic pain syndromes are increasing and particularly in SFN patients, several genetic variants were found mainly located in genes encoding voltage-gated sodium channels. Although up to 30% of SFN patients show genetic alterations, most of these remain of “unknown pathogenic significance” and little is known about “genetic SFN”.

**Objectives:**

The study aimed to determine clinical characteristics of SFN patients carrying a rare genetic variant of unknown significance in pain-associated genes.

**Materials and methods:**

From 2015 to 2020, 66 patients with primarily idiopathic SFN were examined and rare gene variants of unknown significance detected in 13/66 (20%) of these. A detailed medical history with focus on pain was recorded and patients filled in standardized questionnaires to assess physical and emotional burden due to pain.

**Results:**

The authors found 13/66 (20%) patients with rare variants of unknown significance located in pain-associated genes who reported pain refractory to analgesic treatment, a higher number of external factors influencing clinical symptoms, and a higher level of physical impairment and emotional stress due to pain compared with patients without such genetic variants.

**Conclusions:**

Early genetic assessment is recommended to optimize the management of patients with potentially hereditary SFN. Early access to rehabilitation and mental support as well as a consequent elimination of external triggering factors should be granted.

## Einführung

Die Small-Fiber-Neuropathie (SFN) beschreibt eine Erkrankung der dünnen sensiblen Aδ- und C‑Nervenfasern. Ihre strukturelle und funktionelle Schädigung resultiert in neuropathischen Schmerzen meist brennender Qualität, Par‑/Dysästhesien, veränderter Thermo‑/Nozizeption und vegetativen Beschwerden. Wenn eine typische Schmerzanamnese besteht und eine Polyneuropathie ausgeschlossen wurde, wird die Erkrankung diagnostiziert bei Vorliegen einer pathologischen Temperatur- und/oder Schmerzwahrnehmung in der klinisch-neurologischen Untersuchung bzw. der quantitativen sensorischen Testung (QST) und/oder bei einer Reduktion bis hin zum Verlust intraepidermaler Nervenfasern in der Hautbiopsie. Dieses Schema orientiert sich an der aktuellen Literatur [1–3], ein diagnostischer Goldstandard ist nicht etabliert.

Ein Großteil der Betroffenen zieht sich die Nervenschädigung durch z. B. metabolisch-toxische Einflüsse wie einen gestörten Glukosemetabolismus, Alkoholkonsum oder im Rahmen von Autoimmunerkrankungen und Infekten zu [4, 5]. PatientInnen mit einer solch erworbenen SFN profitieren von der kausalen Therapie der zugrunde liegenden Erkrankung [6]. Neben der erworbenen Form der SFN können pathogene Varianten in verschiedenen schmerzassoziierten Genen zur Ausbildung einer hereditären Form der Erkrankung beitragen [7, 8]. Klassischerweise sind hier pathogene *Gain-of-function*-Varianten in den Genen *SCN9A, SCN10A* und *SCN11A*, codierend für die spannungsabhängigen Natriumkanäle NaV1.7, NaV1.8 und NaV1.9 („voltage-gated sodium channel“ [NaV]), zu nennen [9]. Daneben stehen weitere schmerzassoziierte Gene wie *TRPA1, TRPV1* und *TRPV3* (Transient-receptor-potential[TRP]-Kanäle) im Verdacht, eine Rolle bei der Ausbildung einer SFN spielen zu können [10]. Ebenso ist eine symptomatische Überschneidung der SFN mit Erkrankungen aus dem Spektrum der hereditären sensorischen und autonomen Neuropathien (HSAN) möglich, hierbei vorrangig verursacht durch pathogene Sequenzvarianten in den Genen *SPTLC1* und *SPTLC2,* involviert in den Sphingolipidmetabolismus [11]. Elektrophysiologisch werden die neuronale Hyperexzitabilität und somit die Symptomatik durch ein angehobenes Ruhemembranpotenzial, eine erhöhte Aktionspotenzialfrequenz und Spontanentladungen der Neurone generiert [12]. Es sind bislang kaum Optionen zur gezielten Therapie der genetisch veränderten Proteine verfügbar. Wie auch bei den PatientInnen mit idiopathischer Form steht bei hereditärer SFN daher die Symptomkontrolle durch eine antineuropathisch wirksame Medikation im Vordergrund [13]. Diese erreichen trotz Anwendung in einem multimodalen Therapiekonzept nur selten eine erfolgreiche Schmerzlinderung.

Bei der Hälfte der PatientInnen mit Small-Fiber-Neuropathie (SFN) findet sich für die Symptomatik keine erklärende Ätiologie. Diese heterogene Gruppe wird als „idiopathisch“ zusammengefasst. In dieser Gruppe gibt es eine Subgruppe, bei der eine genetische Testung zu einem „Befund“ geführt hat, der zum Zeitpunkt der Diagnostik häufig (noch) keine sicher pathogene genetische Variante in einem schmerzassoziierten Gen aufdeckt. In wenigen Fällen können die Varianten im Verlauf durch Segregationsanalysen oder geeignete funktionelle Analysen hinsichtlich ihrer Pathogenität näher untersucht werden. Meist bleibt die Einordnung der Varianten jedoch weiterhin unklar. Die betroffenen PatientInnen werden in der Regel nicht weiter beobachtet, sodass über ihre klinischen Merkmale wenig bekannt ist, obwohl sie in der klinischen Praxis eine wichtige und herausfordernde Subgruppe bilden. Ziel unseres Projekts ist es, PatientInnen aus dieser Subgruppe zu charakterisieren.

## Kollektiv und Methodik

**Abb. 1:**
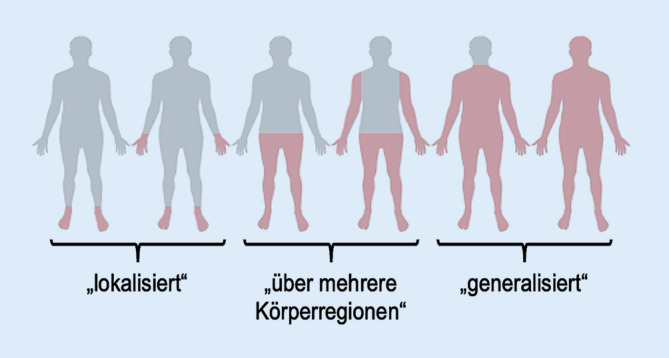
Einteilung der Symptomausbreitung

Von 2015 bis 2020 wurden 66 PatientInnen mit Symptomatik einer SFN untersucht und ihre klinischen Daten rezent veröffentlicht [3]. Nach Ausschluss derer mit erworbener Genese und/oder Großfaserpathologie ist bei den verbleibenden PatientInnen eine Paneldiagnostik auf 28 schmerzassoziierte Gene erfolgt (*AAAS, ARL6IP1, ATL1, ATL3, CLTCL1, DMNT1, DST, FAM134B, FLVCR1, GLA, GMPPA, IKBKAP, KIF1A, NAGLU, NGF, NTRK1, PRDM12, RAB7A, SCN9A, SCN10A, SPTLC1, SPTLC2, TRPA1, TRPM3, TRPV3, TTR *und *WNK1*; [3]). Neben einer detaillierten Symptom‑, Sozial- und Familienanamnese wurden zur Einschätzung von Beschwerdepräsentation und resultierender Belastung mehrere Fragebögen erhoben. Die Schmerzintensität wurde im Neuropathic Pain Symptom Inventory (NPSI, [14]) auf einer numerischen Rating-Skala (NRS) von 0 („kein Schmerz“) bis 10 („maximaler Schmerz“) bewertet. Mit der Graded Chronic Pain Scale (GCPS, [15]) wurde gleichförmig die symptombedingte Limitation in Alltag, Freizeit und Beruf angegeben. Anhand der Allgemeinen Depressionsskala (ADS) wurde in 20 Fragen zu psychischen und somatischen Beschwerden bewertet von 0 („nie“) bis 3 („häufig“) die Depressivität analysiert – ein Score von ≥ 22 Punkten galt als hinweisend auf eine depressive Störung [16]. Die Symptomausbreitung wurde in 3 Gruppen eingeteilt (Abb. 1). Die statistische Auswertung der Daten erfolgte mit SPSS Statistics (IBM, Ehningen, Deutschland). Nach Prüfung auf Normalverteilung mittels Shapiro-Wilk-Test erfolgten vergleichende Analysen normalverteilter Daten mittels t‑Test und nichtnormalverteilter Daten mittels Mann-Whitney-U-Test. Die Testung auf Unabhängigkeit erfolgte mittels Chi-Quadrat-Test bei zu erwartenden Häufigkeiten > 5 und mittels Fisher-Test bei < 5. Bei *p*-Werten < 0,05 wurde in einem explorativen Charakter Signifikanz angenommen.

## Ergebnisse

Das Studienkollektiv bestand aus 27/66 (41 %) Männern und 39/66 (59 %) Frauen. Das mediane Alter der PatientInnen lag bei 50 Jahren (19–78 Jahre). Bei 13/66 (20 %) PatientInnen konnten seltene Varianten in schmerzassoziierten Genen detektiert werden. Die Einordnung der Pathogenität dieser nach Kriterien des American College of Medical Genetics and Genomics (ACMG; [17]) und ihrer Funktion ist in Tab. 1 aufgeführt. Insgesamt wurden 12/13 Varianten als „Variante unklarer Signifikanz“ (VUS) und 1/13 als „wahrscheinlich benigne“ eingestuft.

**Tab. 1:** Detektierte genetischeVarianten im Studienkollektiv

ID	Gen	Proteinfunktion	Variante	Allelfrequenz (%)	ACMG-Klasse
1004	*SCN10A*	Kanalprotein Na_V_1.8 zur Signaltransduktion in Aδ- und C‑Fasern	NM_006514.4:c.1094C > A, p.(Thr365Asn)	0,0011946	VUS
1018	*NGF*	Neuronaler Wachstumsfaktor	NM_002506.3:c.608C > T, p.(Thr203Met)	0,00039769	VUS
1057	*TRPV3*	TRP-Kanalprotein zur Wahrnehmung noxischer Reize	NM_145068.4:c.958A > G, p.(Met320Val)	0,11277	VUS
1069	*TRPM3*	Analog *TRPV3*	NM_206944.4:c.4337T > C, p.(Ile1446Thr)	0,0063719	VUS
1084	*SCN11A*	Kanalprotein Na_V_1.9 zur Signaltransduktion in Aδ- und C‑Fasern	NM_001349253.2:c.603T > G, p.(Ile201Met)	–	VUS
1095	*SCN11A*	Kanalprotein Na_V_1.9 zur Signaltransduktion in Aδ- und C‑Fasern	NM_001349253.2:c.1043C > A, p.(Ser348Tyr)	–	VUS
1096	*SCN9A*	Kanalprotein Na_V_1.7 zur Signalgeneration in Aδ- und C‑Fasern	NM_002977.3:c.4942G > A, p.(Ala1648Thr)	0,00079531	VUS
1097	*TRPA1*	Analog *TRPV3*	NM_007332.3:c.1678C > G, p.(His560Asp)	–	VUS
1102	*SCN10A*	Kanalprotein Na_V_1.8 zur Signaltransduktion in Aδ- und C‑Fasern	NM_006514.4:c.5216_5217delinsTT, p.(Asp1739Val)	–	VUS
1104	*FBLN5*	Fibulin‑5; extrazelluläres Matrixprotein	NM_006329.4:c.212G > A, p.(Arg71GIn)	0,0020082	VUS
1119	*SCN9A*	Kanalprotein Na_V_1.7 zur Signalgeneration in Aδ- und C‑Fasern	NM_002977.3:c.3911T > C, p.(Ile1304Thr)	0,0053484	VUS
1121	*SPTLC1*	Bestandteil des Sphingolipidmetabolismus	NM_006415.4:c.250A > G, p.(Ile84Val)	0,0042914	Wahrscheinlich benigne
1130	*SCN10A*	Kanalprotein Na_V_1.8 zur Signaltransduktion in Aδ- und C‑Fasern	NM_006514.4:c.3417G > C, p.(Trp1139Cys)	0,0099003	VUS

*TRP* „transient receptor potential“, *VUS* Variante unklarer Signifikanz

In 7/13 Fällen lagen die VUS in Genen der spannungsabhängigen Natriumkanäle *SCN9A *(2/7), *SCN10A* (3/7) und *SCN11A* (2/7). 3/13 PatientInnen zeigten je eine Variante in *TRP*-Kanälen: *TRPA1, TRPV3* und *TRPM3*. In je einem Fall konnten Varianten in den Genen *FBLN5, NGF* und *SPTLC1* gefunden werden.

Diese Kohorte mit seltener Variante in schmerzassoziierten Genen (folgend Kohorte 1) unterschied sich in Geschlechterverteilung (*p* = 0,29), Alter (*p* = 0,24) und Body-Mass-Index (*p* = 0,93) nicht von den übrigen 53 PatientInnen mit idiopathischer SFN ohne Nachweis einer Sequenzvariante in den untersuchten Genen (folgend Kohorte 2). Zunächst beschrieben beide Kohorten einen seit dem mittleren Lebensalter bestehenden brennenden Schmerz mäßiger bis hoher Intensität begleitet von Kribbeln und/oder Taubheitsgefühl. Die Symptome waren bei ca. der Hälfte am gesamten Körper ausgeprägt und traten regelhaft als Dauerschmerz mit Spitzen auf. Autonome Symptome, meist Veränderungen der Schweißproduktion, waren bei 49/66 (74 %) der PatientInnen prominent (Tab. 2). Zwischen den Kohorten konnte bei der Symptomausdehnung kein Unterschied gesehen werden (*p* = 0,12), es schilderte aber keine PatientIn der Kohorte 1 eine rein akrale Ausbreitung gegenüber einem Viertel derer in Kohorte 2. Auch das zeitliche Auftreten zeigte tendenziell Unterschiede: Schmerzattacken mit/ohne Dauerschmerz gaben 12/13 (92 %) der PatientInnen in Kohorte 1 und 35/53 (66 %) in Kohorte 2 an (*p* = 0,09). Einflussfaktoren, die Symptome verstärken oder lindern, wurden von allen PatientInnen der Kohorte 1 und 37/53 (70 %) der Kohorte 2 genannt (*p* = 0,03, Tab. 3).

**Tab. 2:** Symptommanifestation im Kohortenvergleich

	SFN	Kohorte 1	Kohorte 2	*p*
*n*	66	13	53	–
Alter bei Beginn, Jahre	44 (8–74)	44 (29–57)	45 (8–74)	0,66
Schmerzintensität, aktuell, auf NRS	4/10 (0–8)	4/10 (1–7)	4/10 (0–8)	1,00
Schmerzintensität, maximal, auf NRS	8,0/10 (3–10)	8,0/10 (4–10)	8,0/10 (3–10)	0,87
**Symptomausbreitung**				0,12
*Lokalisiert*	13/66 (20 %)	0/13 (0 %)	13/53 (25 %)	
*Ausgedehnt*	21/66 (32 %)	6/13 (46 %)	15/53 (28 %)	
*Generalisiert*	32/66 (48 %)	7/13 (54 %)	25/53 (47 %)	
Autonome Symptome	49/66 (74 %)	8/13 (62 %)	41/53 (77 %)	0,29

*NRS* numerische Rating-Skala, *SFN* Small-Fiber-Neuropathie

**Tab. 3:** Einflussfaktoren im Kohortenvergleich

	SFN	Kohorte 1	Kohorte 2	*p*
*n*	66	13	53	–
**Positive Anamnese zu Einflussfaktoren**	50/66 (76 %)	13/13 (100 %)	37/53 (70 %)	0,03*
*Symptomzunahme*	46/66 (71 %)	13/13 (100 %)	34/53 (64 %)	0,01*
*Symptomlinderung*	29/66 (44 %)	10/13 (77 %)	19/53 (36 %)	< 0,01**
**Symptomzunahme**				
*Kälte*	13/66 (20 %)	5/13 (38 %)	8/53 (15 %)	0,11
*Wärme*	21/66 (32 %)	2/13 (15 %)	19/53 (36 %)	0,20
*Druck/Berührung*	34/66 (52 %)	8/13 (62 %)	26/53 (49 %)	0,42
**Symptomlinderung**				
*Kälte*	8/66 (12 %)	1/13 (8 %)	7/53 (13 %)	1,0
*Wärme*	7/66 (11 %)	4/13 (31 %)	3/53 (6 %)	0,03*
*Ruhe*	12/66 (18 %)	4/13 (31 %)	8/53 (15 %)	0,23
*Bewegung*	9/66 (14 %)	4/13 (31 %)	5/53 (9 %)	0,07

*SFN* Small-Fiber-Neuropathie

**Abb. 2:**
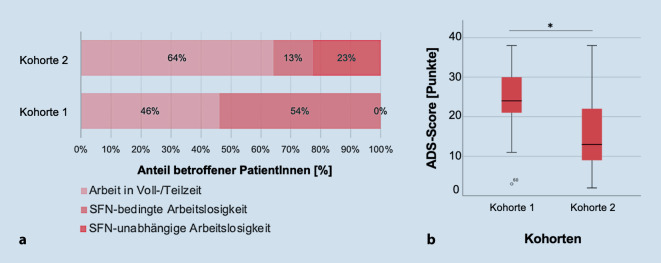
Physische und psychische Limitationen. **a** Erwerbsstatus im Kohortenvergleich. **b** Boxplot-Diagramm des ADS-Scores im Kohortenvergleich

Die Symptomlast zwang 14/66 (21 %) der PatientInnen zur Aufgabe ihrer Erwerbstätigkeit im Sinne einer Krankschreibung, Arbeitslosigkeit oder Berentung. Im Kohortenvergleich war dies bei PatientInnen mit seltener Variante in schmerzassoziierten Genen im höheren Maß notwendig (*p* < 0,01, Abb. 2a). Ihre Limitation im Beruf nach GCPS lag im Mittel höher als in Kohorte 2 (*p* = 0,04). Parallel fiel ein höheres Maß an Depressivität dieser Kohorte auf: Gemessen an der ADS fanden sich 9/13 (69 %) PatientInnen der Kohorte 1 mit behandlungswürdiger Depression gegenüber 14/53 (26 %) in Kohorte 2 (*p* < 0,01, Abb. 2b).

**Abb. 3:**
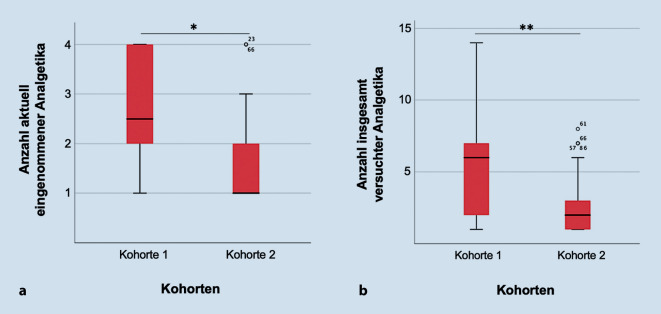
Antineuropathisch wirksame Medikation im Kohortenvergleich. Anzahl: **a** aktuell eingenommene antineuropathische Wirkstoffe, **b** insgesamt versuchte antineuropathische Wirkstoffe. *p < 0,05, **p < 0.01

42/66 (64 %) PatientInnen standen bei Einschluss unter antineuropathisch wirksamer Medikation. Es zeigte sich kein Unterschied im aktuellen Therapieerfolg: Eine Symptomlinderung von > 30 % wurde bei 5/10 (50 %) pharmakologisch behandelten PatientInnen in Kohorte 1 und 22/32 (69 %) in Kohorte 2 erreicht (*p* = 0,23). Dafür war bei PatientInnen ohne Varianten in schmerzassoziierten Genen eine geringere Anzahl an Wirkstoffen notwendig (*p* = 0,02) und es mussten weniger Präparate bis zur Linderung getestet werden (*p* < 0,01, Abb. 3). Einen Überblick über die angewendeten Präparate gibt Tab. 4. Zur antineuropathischen Medikation der 1. Wahl gehören nach Empfehlung der S2k-Leitlinie zur Behandlung des neuropathischen Schmerzes die Wirkstoffe Pregabalin, Gabapentin, Duloxetin und Amitriptylin. Andere anfallssupprimierende, antidepressive Wirkstoffe, Opioide, nichtsteroidale Antirheumatika und u. a. Topika wie Lidocain und Capsaicin gelten als Zweitlinientherapie oder werden nicht empfohlen [13].

**Tab. 4:** Angewendete antineuropathische Wirkstoffe bei Erhalt einer Therapie

	SFN	Kohorte 1	Kohorte 2
*n*	42	10	32
**Erstlinientherapie**	35/42 (83 %)	9/10 (90 %)	26/32 (81 %)
*Symptomlinderung*	29/35 (83 %)	6/9 (67 %)	23/26 (88 %)
*Kein Effekt*	6/35 (17 %)	3/9 (33 %)	3/26 (12 %)
**Zweitlinientherapie/nicht empfohlen**	16/42 (38 %)	7/10 (70 %)	9/32 (28 %)
*Symptomlinderung*	15/16 (94 %)	6/7 (86 %)	9/9 (100 %)
*Kein Effekt*	1/16 (6 %)	1/7 (14 %)	0/9 (0 %)

Einteilung gemäß S2k-Leitlinie zur Behandlung neuropathischer Schmerzen

6/13 (46 %) der PatientInnen aus Kohorte 1 gaben eine Häufung ähnlicher Symptome in ihrer Familie an, gegenüber 11/53 (21 %) ohne Varianten in schmerzassoziierten Genen (*p* = 0,08). Unter genauer Betrachtung der Kohorte 2 zeigte sich, dass mit der positiven Familienanamnese hier auch eine großflächigere Schmerzausbreitung einherging (*p* = 0,02). Zudem gaben diese PatientInnen bei familiärer Häufung auch deutlich häufiger symptomlindernde Einflussfaktoren an (*p* = 0,04).

## Diskussion

Alle PatientInnen des Kollektivs schilderten auf den ersten Blick eine das typische Bild einer SFN wiedergebende Symptomatik: Brennschmerz begleitet von Par‑, Dysästhesien und/oder autonomen Beschwerden. Bei allen diesen PatientInnen war die umfangreiche Ursachenabklärung [3] unergiebig, sodass sie als PatientInnen mit idiopathischer SFN eingestuft wurden. Nach Erhalt der Ergebnisse der genetischen Paneldiagnostik ließen sich zwei Kohorten definieren: Ein Fünftel unserer PatientInnen wies seltene Varianten in schmerzassoziierten Genen auf.

Eine familiäre Häufung der Symptomatik konnte unter diesen PatientInnen nur als Trend dargestellt werden, wobei auch in Kohorte 2 bei positiver Familienanamnese eine durch äußere Faktoren beeinflusste Symptomatik beschrieben wurde. Diese auch meist in Attacken auftretenden Schmerzen bei mutmaßlich genetischer SFN-Ätiologie ähneln der klinischen Präsentation anderer genetisch-neuropathischer Schmerzsyndrome wie der familiären episodischen Schmerzstörungen Typ 1–4 [18] bedingt durch Varianten in den Genen *TRPA1, SCN9A, SCN10A, SCN11A* oder der durch pathogene *SCN9A*-Varianten vermittelten paroxysmalen extremen Schmerzstörung und primären Erythromelalgie [19]: Die Betroffenen schildern in allen Fällen durch äußere Faktoren wie thermische oder mechanische Reize getriggerte Schmerzattacken.

Auch ohne Kenntnis über die mögliche genetische Ätiologie ihrer Erkrankung gaben die PatientInnen der Kohorte 1 eine höhere psychische Belastung an, die zu einem Teil auf die herausfordernde Therapie zurückzuführen war. Zum aktuellen Zeitpunkt erfährt die Behandlung einer genetisch bedingten SFN wenig öffentliches Interesse [20]. Die Forschung an zielgerichteten Therapeutika zur Blockade fehlregulierter Proteine, zum Beispiel spannungsabhängiger Natriumkanäle [21], ist entsprechend sehr wichtig und könnte die Behandlung betroffener PatientInnen maßgeblich verbessern. Eine niederländische Studie untersuchte den Effekt des Natriumkanalblockers Lacosamid bei SFN-PatientInnen mit *Gain-of-function*-Mutation im *SCN9A*-Gen [22]. Hier zeigte sich verglichen mit einem Placebopräparat eine Verbesserung von Schmerzen und Schlafqualität bei Einnahme von Lacosamid. Parallel könnten unter Kenntnis der höheren physischen und psychischen Belastung der SFN-PatientInnen mit seltenen Varianten in schmerzassoziierten Genen frühzeitig Maßnahmen zur Rehabilitation, Wiedereingliederung und Stärkung der Krankheitsbewältigung getroffen werden. Dies kann die klinische Versorgung im Rahmen des bei SFN wichtigen multimodalen Therapiekonzepts optimieren.

### Gendiagnostik bei SFN: Wie, wann und warum?

**Abb. 4:**
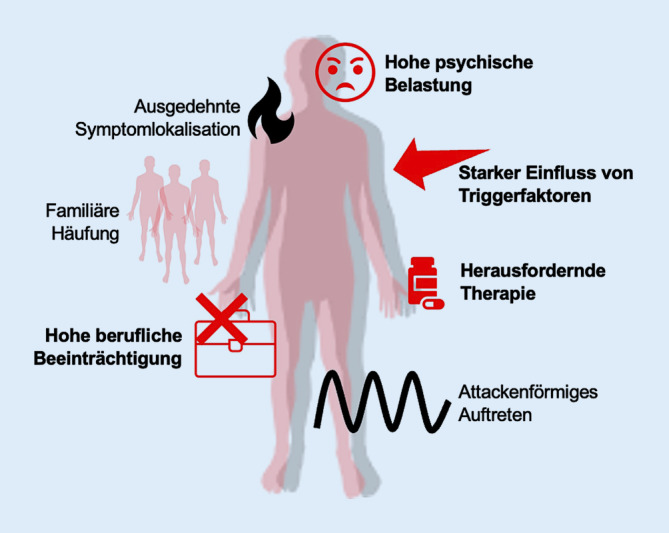
Charakteristika bei SFN-PatientInnen mit seltener Variante in schmerzassoziierten Genen. *Rot* signifikante Unterschiede gegenüber Kohorte 2. *Schwarz* nichtsignifikante, tendenzielle Unterschiede gegenüber Kohorte 2

In der genauen Datenauswertung wiesen die PatientInnen mit seltenen Varianten in schmerzassoziierten Genen subtile Unterschiede zum restlichen Kollektiv auf (Abb. 4). Dennoch konnte auf Basis der erhobenen Daten kein sicheres Kriterium detektiert werden, das bei der Entscheidung für oder gegen eine genetische Diagnostik herangezogen werden könnte. Nur auf Basis dieser Kriterien sollte keinem Patienten eine genetische Diagnostik verwehrt werden. Sie ist insbesondere einzuleiten, wenn andere häufige Ursachen einer SFN ausgeschlossen wurden und eine familiäre Häufung der Symptome vorliegt. Entsprechend der Datenlage zu genetischen Schmerzsyndromen sollten vorrangig Gene der spannungsabhängigen Natriumkanäle *SCN9A, SCN10A* und *SCN11A* und der TRP-Kanäle *TRPA1, TRPV1* und *TRPV3* untersucht werden. Eine ergänzende Analyse der HSAN-assoziierten Gene bietet sich ebenfalls an. Insgesamt fällt der Anteil der PatientInnen, bei denen eine klare monogenetische Ursache der Erkrankung nachgewiesen werden kann, jedoch gering aus. Die große Mehrheit der gefundenen Veränderungen ist in ihrer Einordnung weiterhin unklar. Je weiter die Datenlage genetischer Veränderungen in SFN-Patienten und Kontrollkollektiven in Zukunft ausgebaut wird, desto besser lassen sich Varianten hinsichtlich ihrer Pathogenität einordnen. Ob die beschriebenen klinischen Kriterien tatsächlich Bestand haben, bleibt in größeren Kollektiven herauszufinden. Diese bieten zudem die Möglichkeit der Identifizierung genetischer Veränderungen, die als prädisponierende Faktoren fungieren. Zur Entschlüsselung einer möglichen Wechselwirkung verschiedener Veränderungen sowie bislang unbekannter Pathomechanismen sollte die Datenerhebung im Forschungskontext zudem nicht auf bislang bekannte krankheitsassoziierte Gene beschränkt werden, vielmehr sollten breite Screeningverfahren zur Anwendung kommen.

## Limitationen

Die Anzahl der PatientInnen mit seltener Variante in schmerzassoziierten Genen in unserer Studie ist mit *n* = 13 gering. Zudem handelt es sich um eine heterogene Kohorte, deren Vielzahl gefundener Genvariationen bisher nicht als pathogenetisch relevant bestätigt werden konnte. Neben der genetischen Veränderung sind auch weitere Faktoren wie Komorbiditäten und Lebensumstände an der Pathogenese beteiligt und können die Symptomatik und die Resilienz der Betroffenen modulieren. Das Gleiche gilt für (Neben‑)Wirkungen eingenommener antineuropathischer Wirkstoffe. Solchen Störgrößen wurde durch Anwendung strukturierter Fragebögen entgegengewirkt. Die statistische Analyse unserer Ergebnisse hat einen explorativen Charakter.

## Fazit für die Praxis

- PatientInnen mit SFN, und scheinbar gerade die Subgruppe mit potenziell genetischer Ätiologie, sind in multiplen Lebensbereichen stark belastet, im Besonderen beruflich und psychisch.
- Eine frühe Integration der genetischen Paneldiagnostik in die ätiologische Abklärung einer SFN hilft, diese stark belasteten PatientInnen frühzeitig zu erkennen und in einem multimodalen Therapiekonzept zu unterstützen.
- Im Verlauf sind ggf. zielgerichtete Therapien für Betroffene verfügbar.
